# Model spread in tropical low cloud feedback tied to overturning circulation response to warming

**DOI:** 10.1038/s41467-022-34787-4

**Published:** 2022-11-19

**Authors:** Kathleen A. Schiro, Hui Su, Fiaz Ahmed, Ni Dai, Clare E. Singer, Pierre Gentine, Gregory S. Elsaesser, Jonathan H. Jiang, Yong-Sang Choi, J. David Neelin

**Affiliations:** 1 Department of Environmental Science, University of Virginia, Charlottesville, VA USA; 2 Joint Institute for Research in Earth Systems Science and Engineering, University of California, Los Angeles, Los Angeles, CA USA; 3 Department of Atmospheric and Oceanic Sciences, University of California, Los Angeles, Los Angeles, CA USA; 4 Department of Civil and Environmental Engineering, The Hong Kong University of Science and Technology, Hong Kong SAR, China; 5 Department of Environmental Science and Engineering, California Institute of Technology, Pasadena, CA USA; 6 Earth and Environmental Engineering, Columbia University, New York, NY USA; 7 Department of Applied Physics and Applied Mathematics, Columbia University, New York, NY USA; 8 NASA Goddard Institute for Space Studies, New York, NY USA; 9 Jet Propulsion Laboratory, California Institute of Technology, Pasadena, CA USA; 10 Department of Climate and Energy Systems Engineering, Ewha Womans University, Seoul, South Korea

**Keywords:** Atmospheric dynamics, Climate and Earth system modelling

## Abstract

Among models participating in the Coupled Model Intercomparison Project phase 6 (CMIP6), here we show that the magnitude of the tropical low cloud feedback, which contributes considerably to uncertainty in estimates of climate sensitivity, is intimately linked to tropical deep convection and its effects on the tropical atmospheric overturning circulation. First, a reduction in tropical ascent area and an increased frequency of heavy precipitation result in high cloud reduction and upper-tropospheric drying, which increases longwave cooling and reduces subsidence weakening, favoring low cloud reduction (Radiation-Subsidence Pathway). Second, increased longwave cooling decreases tropospheric stability, which also reduces subsidence weakening and low cloudiness (Stability-Subsidence Pathway). In summary, greater high cloud reduction and upper-tropospheric drying (negative longwave feedback) lead to a more positive cloud feedback among CMIP6 models by contributing to a greater reduction in low cloudiness (positive shortwave feedback). Varying strengths of the two pathways contribute considerably to the intermodel spread in climate sensitivity.

## Abstract (web summary)

The magnitude of the tropical low cloud feedback, which contributes considerably to uncertainty in estimates of climate sensitivity, is closely linked to tropical deep convection and its effects on the tropical atmospheric overturning circulation.

## Introduction

Across generations of climate model intercomparisons, uncertainty in estimates of the projected warming in response to increasing greenhouse gases has persisted^1,2^. While tremendous progress has been made in modeling the Earth system in individual climate models, our ability to narrow the intermodel spread in equilibrium climate sensitivity (ECS), the equilibrium response in global-mean surface temperature to a doubling of carbon dioxide, is still limited. Critical to making significant progress in reducing such uncertainty is explaining the intermodel spread in the strength of the cloud feedback.

Low cloud changes have long been a root cause of uncertainty in ECS through their radiative effects^3–14^. Low-level clouds efficiently reflect incoming solar radiation back to space while only weakly reducing the longwave emission of terrestrial radiation to space, thereby exerting a strong cooling effect on the planet. A decrease in low cloud fraction (LCF) or cloud optical depth with warming would amplify the positive radiative forcing from increasing greenhouse gases by allowing more solar radiation to reach Earth’s surface^15^, constituting a positive feedback.

The latest generation of models participating in the coupled model intercomparison project Phase 6 (CMIP6) exhibit a wider range and higher multi-model-mean ECS than CMIP5 models^2^. The upward shift in ECS can be traced back to stronger positive low cloud feedback in the extratropics^2^, while the tropical (30°S-30°N) low cloud feedback in both trade cumulus and stratocumulus regimes continues to be a dominant source of intermodel differences in ECS^16,17^. Moreover, Zelinka et al.^18^ found that the feedbacks with largest uncertainties in expert assessments and the largest bias across the CMIP6 ensemble are from tropical marine low cloudiness and tropical anvil cloud area. In this study, we consider whether the uncertainty in tropical marine low cloud and anvil cloud area feedbacks might be physically related to one another.

Much work over the last few decades has focused on understanding local factors controlling LCF in large-scale descent regions– such as estimated inversion strength (EIS)^19^, local sea surface temperature (SST)^20^, lower tropospheric stability (LTS)^21^, lower free troposphere relative humidity (RH)^22^, subsidence strength^23^, and the net outgoing longwave radiation (OLR) in the inversion layer^24,25^–particularly in regions of stratocumulus clouds in eastern subtropical ocean basins. Here, however, we consider non-local factors (e.g., deep convection) affecting the local meteorological conditions regulating LCF in the descent region (LCF_d_), defined as the average LCF between 30°S-30°N where the monthly-mean pressure velocity ($\omega$) at 500 hPa is positive. The same definition will be used throughout to define descent regions, and the subscript “d” will be used to denote quantities calculated within these descent regions. We focus on the change of LCF_d_ per degree of global-mean surface temperature change (dLCF_d_/dT_s_) because the tropical (30°S-30°N) net cloud radiative effect (netCRE) change per unit warming from the present-day to the future warmer climate and its relationship to ECS (see also ref. 26) is predominantly contributed by the change in dLCF_d_/dT_s_ (Supplementary Fig. 1). This was also the case in CMIP5 models^12^.

## Results

### Changes to deep convection, clouds, and the overturning circulation

**Fig. 1:**
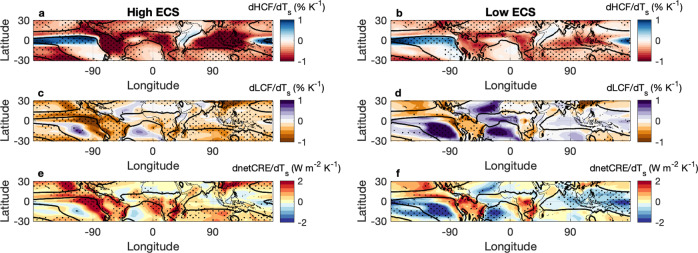
High and low cloud fraction reduction correspond to a more positive net cloud radiative effect and higher climate sensitivity. Composite maps of changes in (**a**, **b**) high cloud fraction (dHCF/dT_s_; % K^−1^), (**c**, **d**) low cloud fraction (dLCF/dT_s_; % K^−1^), and (**e**, **f**) the net cloud radiative effect (dnetCRE/dT_s_; W m^−2^ K^−1^) per degree warming. Changes are calculated as the differences between 2086–2100 (SSP5-8.5) and 2000–2014 (historical). Left and right columns show the composites of high and low climate sensitivity (ECS; K) models, respectively. The 13 highest and 13 lowest ECS models of those used in this study comprise the composites. Stippling represents areas where 9 or more models agree on the sign of the change. The solid lines indicate where *ω*_500_  = 0 in the ensemble mean.

Examining the spatial patterns in cloud changes reveals that the high cloud fraction (HCF) and LCF are dramatically reduced throughout most of the tropics in high ECS models compared to low ECS models (Fig. 1a-d). The patterns of LCF changes most closely resemble patterns in the change in netCRE (Fig. 1e-f), emphasizing the importance of LCF changes to the strength of the total cloud feedback and climate sensitivity (Supplementary Fig. 1). Cloud fraction is interpolated onto 19 pressure levels from the native vertical levels. LCF is computed as the maximum cloud fraction at any level between 600–1000 hPa, assuming maximum overlap. HCF is computed as the maximum cloud fraction at any level between 100–250 hPa (assuming maximum overlap). The greater decrease in HCF per degree warming in the high ECS models, compared to the low ECS models, suggests that detrainment of condensate from deep convection decreases more in high ECS models than low ECS models. This decreased detrainment could result from a variety of factors, including greater tropospheric stability in response to greenhouse gas forcing^27,28^, increases in precipitation efficiency^29^, and/or a reduction in the area occupied by deep convection^30^.

**Fig. 2:**
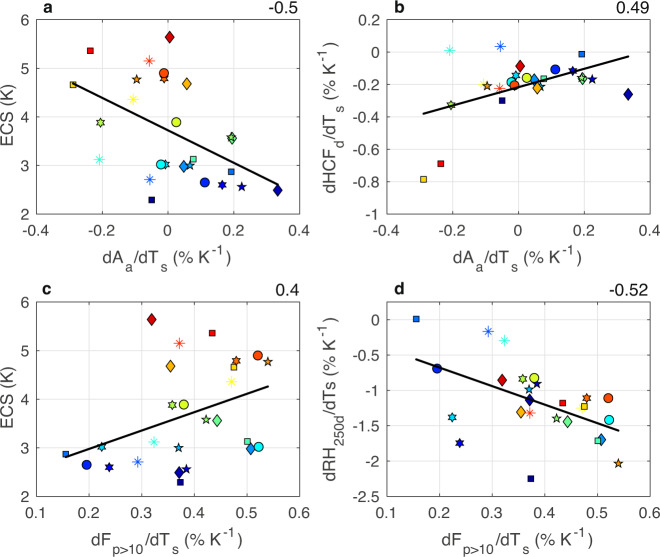
Greater ascent area fraction reduction and increases in the frequency of heavy precipitation are seen in models with higher climate sensitivity. The relationships between (**a**) the change in ascent area fraction (dA_a_/dT_s_; % K^−1^) and climate sensitivity (ECS; K), (**b**) dA_a_/dT_s_ and the change in desent region high cloud fraction (dHCF_d_/dT_s_; % K^−1^), (**c**) the change in the frequency of heavy precipitation (dF_p>10_/dT_s_; % K^−1^) and ECS, and (**d**) dF_p>10_/dT_s_ and the change in descent region upper tropospheric relative humidity (dRH_250d_/dT_s_; % K^−1^) for 26 CMIP6 models. The color scale from blue to red reflects increasing ECS values. All relationships shown are tropical averages (30°S-30°N), including both land and ocean points. Changes are calculated as the differences between 2086–2100 (SSP5-8.5) and 2000–2014 (historical). Values in the upper right are Pearson correlation coefficients for the regression lines shown.

Indeed, higher ECS models show greater reductions of tropical ascent area (A_a_; Fig. 2a), which corresponds to greater decreases in HCF_d_ per degree warming (Fig. 2b). Tropical ascent area is defined as the region of the tropics where ω_500_ is negative from 30°S-30°N. This metric relates to a narrowing of the intertropical convergence zone (ITCZ) in the zonal mean^31–33^. Higher ECS models also show greater increases in the frequency of heavy precipitation (Fig. 2c). In other words, models that exhibit more dramatic regime shifts towards heavier precipitation in a warmer world have a higher ECS. The frequency of heavy precipitation (F_p>10_) is estimated from the frequency of grid boxes in which total precipitation (convective and stratiform) exceeds 10 mm day^−1^ from monthly average values. A greater F_p>10_ probably relates to greater increases in precipitation efficiency and convective organization^34^; a greater F_p>10_ is related to a drier upper troposphere (Fig. 2d), a signature of environments with greater degrees of convective clustering/organization^35^. We acknowledge, however, that we are limited in our interpretation by the monthly output and resolution of the datasets chosen. In summary, high ECS models have greater increases in heavy precipitation events in the future that occupy less total ascent area in comparison to low ECS models, which reduces the detrainment of water vapor and condensate into the upper troposphere. We thus seek a physical explaination for how such a reduction of high cloudiness and detrainment due to changes in deep convection results in a higher climate sensitivity.

### Linking cloud changes to circulation changes

**Fig. 3:**
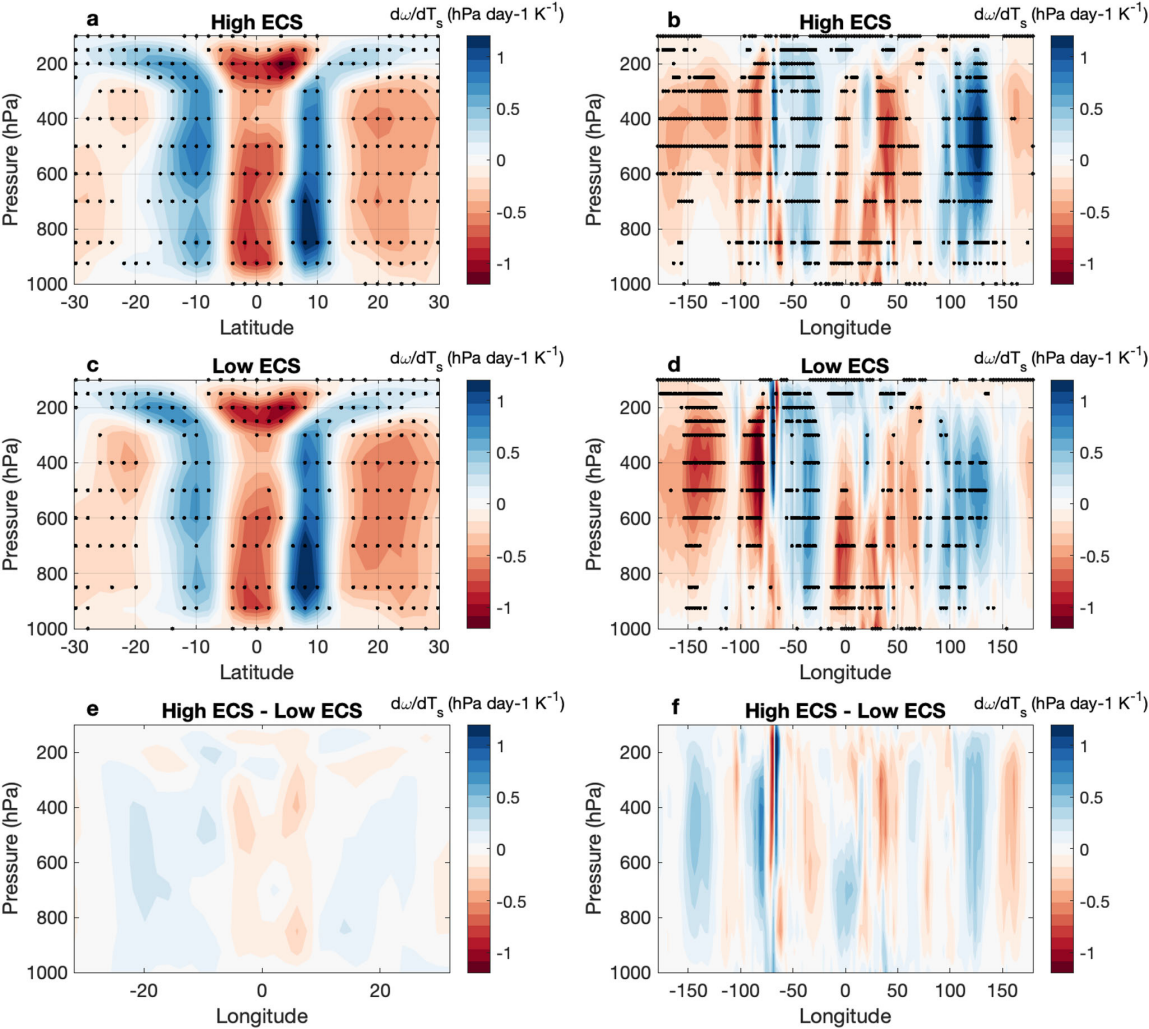
Subsidence generally weakens more in models with a lower climate sensitivity than in models with a higher climate sensitivity. Zonal (**a**, **c**, **e**) and meridional (**b**, **d**, **f**) mean composites (30°S-30°N) of the change in pressure velocity (*ω*; hPa day^−1^ K^−1^) per degree of surface warming (d*ω*/dT_s_) for (**a**, **b**) high climate sensitivity (ECS; K) and (**c**, **d**) low ECS models and the differences between high and low ECS models (**e**, **f**). The 13 highest and 13 lowest ECS models of those used in this study comprise the composites, as in Fig. 1. Stippling indicates where 9 or more models agree on the sign of the change.

**Fig. 4:**
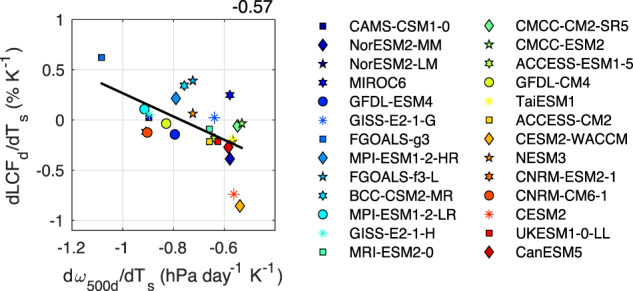
A greater decrease in low cloud fraction corresponds to a lesser reduction of subsidence with warming. The relationship between the change in subsidence rate (d*ω*_500d_/dT_s_; hPa day^−1^ K^−1^) and the change in descent region low cloud fraction (dLCF_d_/dT_s_; % K^−1^) for 26 CMIP6 models. The color scale from blue to red reflects increasing equilibrium climate sensitivity (ECS) values. All relationships shown are tropical averages (30°S-30°N) including both land and ocean points. Changes are calculated as the differences between 2086–2100 (SSP5-8.5) and 2000–2014 (historical). The value in the upper right is the Pearson correlation coefficient for the regression line shown. The relationship is statistically significant at the 95% confidence level (*p* = 0.0025).

We wonder whether interactions between deep convection and the low cloud feedback through changes to the atmospheric overturning circulation may explain this relationship between deep convective changes and ECS. Figures 3a, c, and e show that the subsidence weakens less in the subtropics in high ECS models than in low ECS models. Figures 3b, d, and f show that this is especially true over the Eastern Pacific and Eastern Atlantic. Moreover, the intermodel spread in dLCF_d_/dT_s_ across the tropics (both land and ocean regions considered in the averages) is significantly anti-correlated with the intermodel spread in subsidence strength (Fig. 4). In all CMIP6 models, subsidence strength is projected to decrease with warming, as the increase in dry static stability in response to surface warming dominates over the increase in radiative cooling. Su et al.^36^ also found that all 77 of their analyzed CMIP5 simulations (atmosphere-only and coupled) produced a weakening of subsidence, even despite a variety of surface warming patterns among the coupled model simulations. We thus wonder whether changes to deep convection relate to the response in subsidence rate with warming.

First, let us consider the role of subsidence on low clouds. Strong subsidence generally disfavors low cloudiness in the presence of a strong inversion^23,37^, and thus when subsidence weakens among models, LCF_d_ increases. This seemingly counterintuitive relationship can be explained in terms of dynamical and thermodynamic effects. Dynamically speaking, weaker subsidence permits greater boundary layer growth, which allows stratocumulus clouds to grow higher and thicken, increasing LCF^23^. While this dynamic mechanism would act to cool the planet through a negative shortwave radiative effect, increasing cloud top heights with weaker subsidence would result in infrared emission at cooler temperatures, thus imparting a confounding longwave warming influence. Scott et al.^37^ found that in observations, the warming effect largely cancels the cooling effect, such that the net radiative effect of subsidence changes is small, though the cloud fraction changes (shortwave effect) generally win out over the cloud altitude changes (longwave effect). Overall, the purely dynamical effect is not the primary meteorological control on perturbations to low cloudiness in the tropics.

**Fig. 5:**
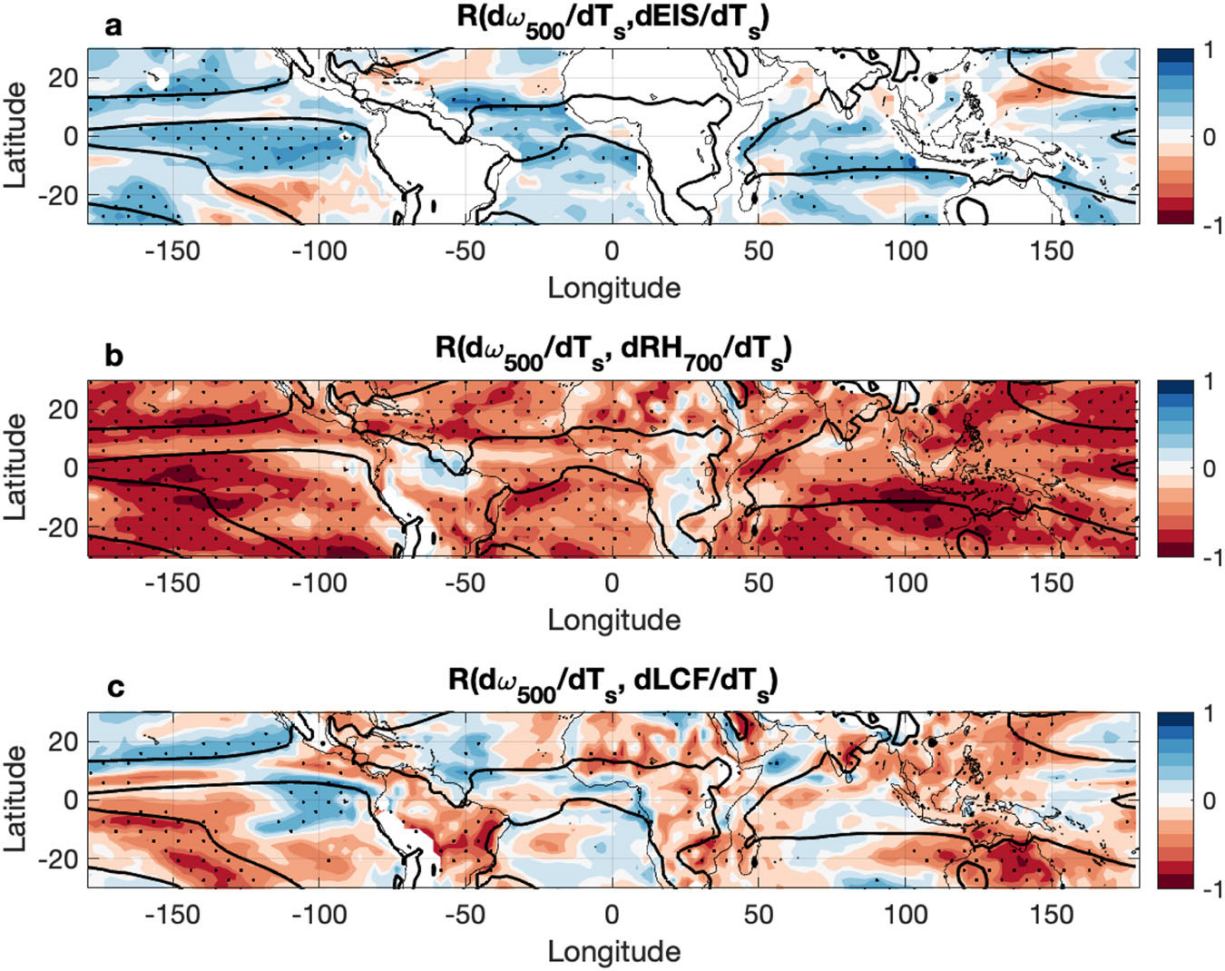
Low cloud fraction decreases most closely correspond to a lesser reduction of subsidence along convective margins where a resulting drier free troposphere may disfavor low cloudiness through entrainment drying. The local Pearson correlation coefficients between (**a**) changes to the pressure velocity at 500 hPa (d*ω*_500_/dT_s_; hPa day^−1^ K^−1^) and changes in the estimated inversion strength (dEIS/dT_s_; K K^−1^), (**b**) d*ω*_500_/dT_s_ (hPa day^−1^ K^−1^) and the change in lower free tropospheric relative humidity (dRH_700_/dT_s_; % K^−1^), and (c) d*ω*_500_/dT_s_ (hPa day^−1^ K^−1^) and the change in low cloud fraction (dLCF/dT_s_; % K^−1^). Stippling indicates that the relationships are statistically significant at 95% (*p* < 0.05) among the 26 models used in the study.

Perturbations to the subsidence rate can greatly modify the humidity and temperature structures of the lower troposphere, however, which are known to have profound influences on low cloudiness across the tropics. Figure 5 illustrates how the intermodel spread in d$\omega$_500_/dT_s_ relates to dEIS/dT_s_ (Fig. 5a) and dRH_700_/dT_s_ (Fig. 5b). EIS is defined as EIS = LTS - Γ_850m_ (Z_700_-LCL), where LTS is the lower tropospheric stability parameter (LTS = θ_700_-θ_surf_), Γ_850m_ is the moist adiabatic potential temperature gradient at 850 hPa, Z_700_ is the altitude of the 700 hPa level, and LCL is the lifting condensation level^19^. There is, indeed, a statistically significant correlation between d$\omega$_500_/dT_s_ and dEIS/dT_s_ throughout a large part of the tropics, especially in regions along convective margins, defined as regions where trade cumulus regimes transition to deep convection on a seasonally varying basis. However, these are not regions in which EIS generally exhibits a strong control on low cloudiness. Instead, EIS is most closely associated with low cloudiness in stratocumulus regimes, where a stronger inversion favors low clouds, and thus the intermodel spread in dEIS/dT_s_ is most closely associated with the intermodel spread in dLCF/dT_s_ in stratocumulus regions (Supplementary Fig. 2a). We, therefore, do not suspect that the effect of d$\omega$_500_/dT_s_ on dEIS/dT_s_ (and thus dLCF/dT_s_) is a primary contributor to the dLCF_d_/dT_s_ relationship to d$\omega$_500d_/dT_s_ (Fig. 4). On the other hand, there is a uniform, tropics-wide statistically significant negative correlation between d$\omega$_500_/dT_s_ and dRH_700_/dT_s_ (Fig. 5b), which suggests that d$\omega$_500_/dT_s_ is closely associated with dRH_700_/dT_s_ everywhere. In descent regions, this negative relationship can be physically interpreted as greater subsidence leading to a drier free troposphere. While the control of RH on low cloudiness differs across different regions of the tropics (Supplementary Fig. 2b), the relationship between circulation and lower free tropospheric humidity is strong, suggesting that dRH_700d_/dT_s_ is likely the primary contributor to the dLCF_d_/dT_s_ relationship to d$\omega$_500d_/dT_s_.

It is worth noting, however, that dRH_700d_/dT_s_ and dLCF_d_/dT_s_ in the descent region are not correlated (R = 0.09) since the correlations between dRH_700_/dT_s_ and dLCF/dT_s_ differ in sign between trade cumulus and stratocumulus regions (Supplementary Fig. 2b). On the one hand, drying disfavors low clouds by enhancing entrainment drying; on the other hand, drying increases radiative cooling at the cloud top, which promotes coupling/mixing of the cloud layer with the surface and favors low clouds^24,38,39^. The cloud-top radiative cooling mechanism is thought to act more strongly over stratocumulus regions, whereas the entrainment drying mechanism is thought to act more strongly over trade cumulus regions, which is consistent with the direction of the signs shown in Supplementary Fig. 2b.

Figure 5c highlights the regions with the strongest local correlations between dLCF/dT_s_ and d$\omega$_500_/dT_s_. These regions include convective margins along the South Pacific Convergence Zone, in the Indo-Pacific region, and along continental convective margins. These are also regions in which dRH_700_/dT_s_ and dLCF/dT_s_ are positively correlated (Supplementary Fig. 2b), suggesting that enhanced entrainment drying along convective margins due to a lesser weakening of subsidence may be a dominant mechanism through which dLCF_d_/dT_s_ reduces in response to circulation changes.

### Energetic constraints on subsidence rate changes

We will now consider how changes to A_a_ and F_p>10_ can physically relate to changes in subsidence. We start by considering the first-order energetic constraint in the tropical descent regions in Eq. (1)^27^:

1 $${\omega }_{{{{{\rm{d}}}}}}\ast {{{{{\rm{S}}}}}}_{{{{{\rm{d}}}}}}={{{{{\rm{F}}}}}}_{{{{{\rm{atm}}}}},d}$$

where F_atm,d_ is the atmospheric cooling rate (F_atm,d_ > 0), ω_d_ is the column-average pressure velocity (subsidence is positive), and S_d_ is the dry static stability, where each quantity is considered to be a column integral. Assuming the column-average subsidence rate *ω*_d_ is proportional to the pressure velocity at 500 hPa (*ω*_500d_) with a scaling factor of $\alpha$ (*ω*_d_ = $\alpha \omega$_500d_) and ignoring the vertical variation of S_d_, we have $\alpha$*ω*_500d_*S_d_ = F_atm,d_. Differentiating with respect to global-mean surface air temperature (T_s_) gives us the change of *ω*_500d_ per degree of surface warming, where F_atm,d_ is signed positive for cooling:

2 $$d{\omega }_{500d}/d{T}_{s}=\left(\frac{1}{\alpha {S}_{d}}\right)d{F}_{atm,d}/d{T}_{s}-({\omega }_{500d}/{S}_{d})\,d{S}_{d}/d{T}_{s}$$

Equation (2) shows that dω_500d_/dT_s_ depends on the responses of F_atm,d_ and S_d_ to T_s_ as well as climatological *ω*_500d_, F_atm,d_ and S_d_.

In the results that follow, we show that the changing properties of deep convection (dA_a_/dT_s_ and dF_p>10_/dT_s_) link directly to dOLR_d_/dT_s_, dS_d_/dT_s_, and S_d_, and thus the low cloud feedback, through their effects on d*ω*_500d_/dT_s_.

### The Radiation-Subsidence Pathway

**Fig. 6:**
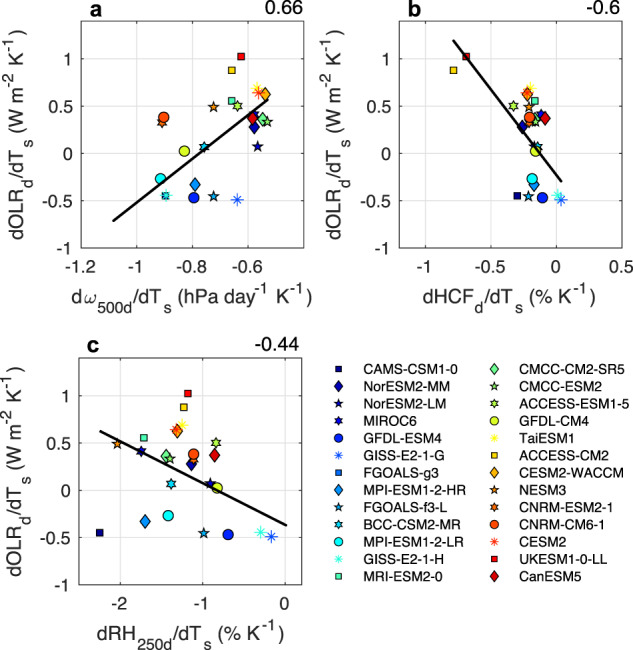
A lesser reduction of subsidence corresponds to a greater increase in outgoing longwave radiation, which is linked to decreased high cloud fraction and upper tropospheric relative humidity. The relationships between (**a**) changes in subsidence rate (d*ω*_500d_/dT_s_; hPa day^−1^ K^−1^) and changes in descent region outgoing longwave radiation (dOLR_d_/dT_s_; W m^-2^ K^−1^), (**b**) changes in the descent region high cloud fraction (dHCF_d_/dT_s_; % K^−1^) and dOLR_d_/dT_s_, and (**c**) changes in descent region upper tropospheric relative humidity (dRH_250d_/dT_s_; % K^−1^) and dOLR_d_/dT_s_ for 26 CMIP6 models. The color scale from blue to red reflects increasing equilibrium climate sensitivity (ECS) values. All relationships shown are tropical averages (30°S-30°N) including both land and ocean points. Changes are calculated as the differences between 2086–2100 (SSP5-8.5) and 2000–2014 (historical). Values in the upper right are Pearson correlation coefficients for the regression lines shown. All relationships are statistically significant at the 95% confidence level.

We start by investigating how dOLR_d_/dT_s_ is modified throughout the tropics, as this relates strongly to the intermodel spread in d*ω*_500d_/dT_s_ (Fig. 6a) and thus dLCF_d_/dT_s_. Figure 6 shows that dOLR_d_/dT_s_ is largely controlled by dHCF_d_/dT_s_ (Fig. 6b), which relates mostly closely with dA_a_/dT_s_ (Fig. 2b). Figure 6c shows that dOLR_d_/dT_s_ is also largely controlled by dRH_250d_/dT_s_, which relates most closely with dF_p>10_/dT_s_ (Fig. 2d). As stated previously, models with greater dF_p>10_/dT_s_ have a drier upper troposphere (UT), possibly due to increased precipitation efficiency and/or convective organization^34^. In summary, when A_a_ reduces and the frequency of heavy precipitation events increases, high clouds reduce, UT RH reduces, OLR increases, subsidence weakens less dramatically, and low clouds reduce. This physical mechanism, which we refer to as the “Radiation-Subsidence Pathway”, constitutes a net positive cloud feedback among CMIP6 models. We suspect that the Radiation-Subsidence mechanism is acting most strongly along seasonally-meandering convective margins in the tropics; the relationship between d*ω*_500_/dT_s_ and dLCF/dT_s_ is strongest in convective margin regions (Fig. 5c) (as are relationships between dω_500_/dT_s_ and dOLR/dT_s_; Supplementary Fig. 3).

In summary, the Radiation-Subsidence Pathway links the intermodel spread in dA_a_/dT_s_ and dHCF_d_/dT_s_, as well as dF_p>10_/dT_s_ and dRH_250d_/dT_s_, to the intermodel spread in dOLR_d_/dT_s_. The model spread in these UT quantities contribute to the model spread in the low cloud feedback in descent regions by modifying the subsidence rate and related thermodynamic cloud controlling factors. Interestingly, a stronger “Iris” effect^40–42^ associated with a greater reduction of HCF_d_ and RH250_d_ (a more negative longwave feedback) ultimately leads to a more positive net cloud feedback in the tropics by contributing to a greater reduction of LCF (a more positive shortwave feedback).

### The Stability-Subsidence Pathway

**Fig. 7:**
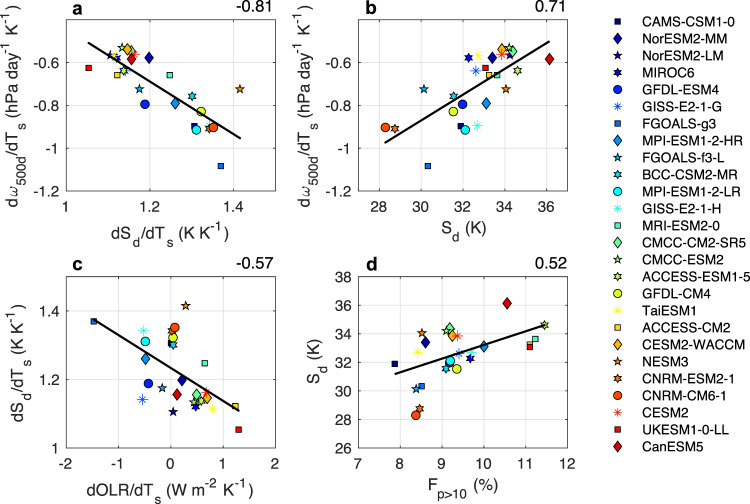
Changes in static stability with warming, as well as a model’s mean-state static stability, are closely associated with subsidence rate changes. The relationship between (**a**) changes in descent region static stability (dS_d_/dT_s_; K K^−1^) and changes in the subsidence rate (d*ω*_500d_/dT_s_; hPa day^−1^ K^−1^), (**b**) descent region mean-state static stability (S_d_; K) and d*ω*500_d_/dT_s_, (**c**) changes in tropics mean outgoing longwave radiation (dOLR/dT_s_; W m^−2^ K^−1^) and dS_d_/dT_s_, and (**d)** the mean-state frequency of heavy precipitation (F_p>10_; %) and S_d_ for 26 CMIP6 models. The color scale from blue to red reflects increasing equilibrium climate sensitivity (ECS) values. All relationships shown are tropical averages (30°S-30°N) including both land and ocean points. Changes are calculated as the differences between 2086–2100 (SSP5-8.5) and 2000-2014 (historical). Values in the upper right are Pearson correlation coefficients for the regression lines shown. All relationships shown are statistically significant at the 95% confidence level.

We now examine how stability (S_d_ and dS_d_/dT_s_) relates to the intermodel spread in d*ω*_500__d_/dT_s_ (Fig. 7a-b). The S_d_ term is calculated as the difference between the mean potential temperature ($\theta$) in the 250–400 hPa layer and $\theta$ in the 700–925 hPa layer. First, if the static stability of the troposphere increases more, the subsidence rate will weaken more drastically (Fig. 7a). Additionally, Fig. 7b illustrates a connection to a model’s climatological static stability: models with a more stable troposphere will have weaker circulation changes in response to greenhouse gas forcing. Both results are consistent with our expectations from Eq. (2). We collectively refer to these mechanisms relating stability to subsidence, and thus low cloudiness, as the “Stability-Subsidence Pathway”.

This exploration leads us to reflect upon what physics modify the stability of the tropical troposphere. Radiative and non-radiative (latent) heating collectively determine the static stability of the troposphere, and thus we wonder whether radiative or latent heating contribute more to the intermodel spread in S_d_ and dS_d_/dT_s_. We explore two proxies for latent and radiative heating– F_p>10_ and OLR, respectively–in each grid cell using monthly mean output.

First, we find that the intermodel spread in dOLR/dT_s_ is related to dS_d_/dT_s_ (Fig. 7c) while dF_p>10_/dT_s_ is not (R = −0.06). Note that here we use the tropical mean OLR, not OLR_d_, as this pathway describes a remote teleconnection between any regions experiencing increases in OLR and its effect on free tropospheric temperatures through wave dynamics (weak temperature gradient theory^43^). Thus, the Stability-Subsidence pathway as it relates dS_d_/dT_s_ to d$\omega$_500d_/dT_s_ can simply be viewed as an extension of the Radiation-Subsidence Pathway. We then find that mean-state F_p>10_ relates most strongly with mean-state S_d_ (Fig. 7d). In other words, the intermodel spread in longwave radiative cooling is the suspected primary driver of the intermodel spread in the change in stability with warming, while the intermodel differences in a model’s mean-state static stability are most closely related to deep convective activity and latent heating.

**Fig. 8:**
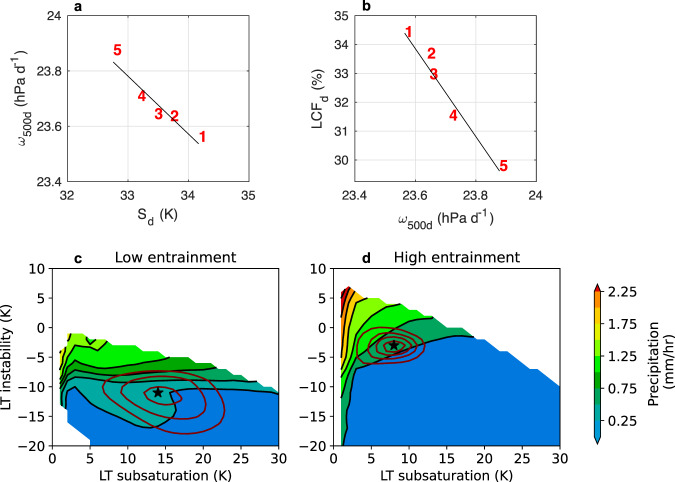
Higher entrainment rates lead to weaker static stability, stronger subsidence, and fewer low clouds than lower entrainment rates. The relationships between (**a**) the descent region pressure velocity at 500 hPa (*ω*_500d_; hPa day^−1^) and the descent region static stability (S_d_; K) and (**b**) *ω*_500d_ and the descent region low cloud fraction (LCF_d_; %) among a CAM5.3 perturbed physics ensemble in which entrainment was perturbed from low to high rates: (1) 0.08 km^−1^ (2) 0.16 km^−1^, (3) 0.25 km^−1^, (4) 0.5 km^−1^, and (5) 1.5 km^−1^. Each value is an average of monthly mean output from a single 11-year run from 1995–2005. All relationships shown are tropical averages (30°S-30°N) including both land and ocean points. The output used to create plots (**a**) and (**b**) are monthly means at a 2 × 2.5 degree resolution. **c** The CAM5.3 precipitation rate (shading) and probability distribution function (PDF) of points with hourly precipitation exceeding 10 mm day^−1^ (maroon contours with intervals of 1 × 10^−3^ and peak contour value of 4 × 10^−3^), shown as functions of lower tropospheric subsaturation and instability (see Methods for definitions). The black star denotes the mode of the precipitating PDF. The CAM5.3 entrainment rate is fixed at 0.125 km^−1^. **d** Same as in (**c**) except for an entrainment rate of 1 km^−1^. Both (**c**) and (**d**) are produced using 3-hourly instantaneous CAM5.3 output at a 0.9 × 1.25 degree resolution.

To further investigate the role of deep convective processes in modifying the static stability of the tropical troposphere, the tropical overturning circulation, and the low cloud feedback, we turn to the output from a perturbed physics ensemble from the Community Atmosphere Model version 5.3 (CAM5.3). Many studies have identified how sensitive the climate system is to the entrainment parameter in climate models^44–49^. Notably, higher entrainment rates yield weaker static stability among ensemble members (Fig. 8a; see also^50,51^). Thus, when entrainment is higher, subsidence is stronger and there are fewer low clouds (Fig. 8b). Perturbing entrainment creates a notable spread in the tropical mean LCF ranging between roughly 29–35% (Fig. 8b).

In summary, the intermodel spread in changes to the static stability of the tropical atmosphere largely affects the intermodel spread in changes to the subsidence strength (Fig. 7a). Moreover, the intermodel spread in dS_d_/dT_s_ is governed in large part by the intermodel spread in dOLR/dT_s_ (Fig. 7c), which is determined by changes to high cloudiness and UT RH (Fig. 6b-c) that are affected by changes to F_p>10_ and A_a_ (Fig. 2b,d). We also find that a model’s mean state static stability matters considerably for how the overturning circulation will respond to warming (Fig. 7b). Factors controlling the onset of deep convection in models, such as the entrainment rate, will inevitably modify the climatological static stability of a given model’s troposphere (Fig. 8). This in turn will modify the tropical overturning circulation response to warming and its effect on the strength of the low cloud feedback. This is just one example of the far-reaching consequences of inadequate observational constraints on deep convective parameterization.

### Further investigation of the Stability-Subsidence and Radiation-Subsidence Pathways

In what follows, we further investigate two key aspects of the Radiation-Subsidence and Stability-Subsidence Pathways. First, we ask ourselves how stability can be modified by entrainment. Then, we explore whether evidence of low cloud reduction exists in additional GCM experiments where OLR is artificially increased. Lastly, we discuss other potential mechanisms linking changes in OLR and static stability to the low cloud feedback.

To explore the role of entrainment in modifying the stability of the tropical troposphere, we use CAM5.3 PPEs with 3-hourly output alongside inferences from the tropical precipitation-buoyancy relationship^52–54^. This relationship suggests that a single value of lower-tropospheric buoyancy (B_L_) separates precipitating and non-precipitating regimes and governs the onset of precipitation at subdaily time scales in observations. This also holds for several CMIP6 models^55^ and is consistent with observations of a regionally independent sub-cloud moist static energy threshold for deep convection at daily timescales in the deep tropics^56^. The precipitation onset determines the most probable thermodynamic phase space for precipitating points^57–59^, thereby governing the mean state. The B_L_ measure is a function of lower-tropospheric measures of subsaturation and convective instability (see Methods); a consequence is that the B_L_ threshold is attained in stable environments when convection’s moisture sensitivity is small but in unstable environments if the moisture sensitivity is large^60^. This behavior is confirmed using CAM5.3 sub-daily output (Fig. 8c-d) in which weak entrainment (Fig. 8c) permits convective onset in a more stable troposphere, compared to a case with strong entrainment (Fig. 8d). The precipitating probability density function (PDF) mode—an implicit measure of the mean state—is accordingly situated in more stable environments when the entrainment is weak. The precipitating PDF is also wider when entrainment is weaker—implying that model convection is more easily triggered and more frequent. Relating back to Fig. 7d, one plausible reason for the correlation between more frequent deep convection and a more stable troposphere is intermodel differences in entrainment rate (or similar parameters governing moisture sensitivity within convective parameterizations). Overall, factors controlling the onset of deep convection in models, such as the entrainment rate, will inevitably modify the static stability of the troposphere. This in turn will modify the tropical overturning circulation response to warming and its effect on the strength of the low cloud feedback.

Finally, additional evidence of a LCF reduction resulting from increased OLR can be seen in two additional sets of simulations: (1) simulations with low and high values of the fall speed of stratiform ice in the Community Atmosphere Model version 5.3 corresponding to different amounts of high clouds in the tropical mean (Supplementary Fig. 4), and (2) atmosphere-only simulations in select participating CMIP6 models available from the Cloud Feedback Model Intercomparison Project (CFMIP) where longwave cloud radiative effects are disabled (Supplementary Fig. 5). As stratiform ice fall speeds increase, there are fewer high clouds and low clouds in the tropics in comparison to the simulations with slower stratiform ice fall speeds, which have greater tropical HCF and LCF (Supplementary Fig. 4). Additionally, in comparing the multi-model mean of simulations with longwave cloud radiative effects disabled (amip-lwoff) to the control simulations (amip), the amip-lwoff experiments show that with increased OLR, LCF decreases uniformly across the tropics (Supplementary Fig. 5).

### Other possible mechanisms linking deep convection changes to low cloud changes

The Radiation-Subsidence and Stability-Subsidence pathways introduced in this study pertaining to circulation changes are unlikely to be the only physical pathways explaining the linkage between decreased high cloudiness and decreased low cloudiness. For instance, changes in radiative heating across the tropics could directly impact the stability of the lower troposphere independent of any influence of circulation changes. As we see from Fig. 6, changes in OLR across the tropics are largely driven by changes in HCF and UT water vapor, both of which are strongly modulated by changes to F_p>10_ and A_a_. By looking at the CFMIP amip-lwoff vs. amip experiments, we find that the LTS decreases throughout the entire tropics, especially in stratocumulus regions, which corresponds to a systematic decline in LCF (Supplementary Fig. 5). One explanation for this response is that the large temperature decrease in the free troposphere in the regions of greatest HCF is communicated through wave dynamics throughout the tropics, according to the weak temperature gradient, decreasing LTS. Overall, dOLR/dTs can affect lower tropospheric stability throughout the tropics, which may also be contributing significantly to the intermodel spread in dLCF_d_/dT_s_ independent of any circulation changes.

There are other potential mechanisms that may be acting to modify the tropical low cloud feedback that relate to convective processes but that, unlike the mechanisms presented in this study, do not necessarily connect to circulation changes or to changes in upper-tropospheric characteristics. For instance, Hirota et al.^61^ found that erroneously active deep convection was related to fewer low clouds in model climatology and led to a reduced low cloud feedback. On a similar note, a local change in deep convective activity along convective margins, perhaps due to changing SST pattern, would directly affect the shallow cloud landscape in those margin regions. Moreover, a direct reduction of detrainment from congestus and deep convection into the lower free troposphere due to increases in precipitation efficiency could reduce cloud fraction through entrainment drying, which might contribute rather directly to a reduction of low cloudiness. However, this would likely depend on the relative rate of drying occurring simultaneously in the boundary layer. For instance, Sherwood et al.^7^ found that enhanced mixing between the boundary layer and lower free troposphere in a warmer climate on both large and small scales leads to a greater reduction of low clouds and a higher ECS among CMIP5 models. Enhanced mixing, in their definition, is a metric relating free tropospheric moistening to boundary layer drying, whereby enhanced mixing would leave the free troposphere more humid and the boundary layer drier. The mixing can be convective in nature (occurring at the sub grid-scale) or at larger, resolved scales along isentropes. Regardless of the scale, the apparent mechanism is that convective mixing dehydrates the boundary layer at a rate that increases as the climate warms. The rate of increase depends on the initial mixing strength, which links the mixing rate in current climate to the tropical low cloud feedback, permitting observational constraints on climate sensitivity.

Finally, it is possible that certain changes to the tropical atmosphere may cause a simultaneous decrease in high cloudiness and low cloudiness without there being a physical linkage among cloud changes. For example, in models with more warming and a greater increase in tropopause height, cloud anvils would be in a more stable environment, which would lead to a decrease in cloud cover due to a decrease in upper-level divergence^27,28^. Meanwhile, because the troposphere is deeper, subsidence would occur over a longer distance, which may lead to lower RH in the lower free troposphere, enhanced cloud-top entrainment drying, and greater mixing of dry air into the boundary layer^62^, thus reducing low cloudiness. In this case, the simultaneous reduction of high and low clouds may both be driven by the deepening of the troposphere, but they would not drive each other directly.

## Discussion

In this work, we find that the CMIP6 model spread in the tropical low cloud feedback is intimately tied to the tropical overturning circulation response to warming. Further, we relate the response of the overturning circulation to deep convective processes. First, we find that models with greater tropical A_a_ reductions and greater increases in the frequency of heavy precipitation under warming tend to have higher ECS. We suggest a causal pathway, whereby reduced HCF_d_ and RH250_d_ leads to increased OLR_d_, resulting in less subsidence weakening and ultimately favoring greater LCF_d_ reduction (the Radiation-Subsidence Pathway).

Additionally, we find that the change in strength of the overturning circulation in response to warming is linked to the low cloud feedback and ECS through the Stability-Subsidence Pathway. The Stability-Subsidence Pathway links subsidence weakening to climatological S_d_ and the response of S_d_ to warming. As an extension from the Radiation-Subsidence Pathway, increased longwave cooling decreases tropospheric stability, which reduces subsidence weakening and low cloudiness. Additionally, we show that the frequency and intensity of tropical deep convection within a model, set largely by its deep convective parameterization, to a large extent determines the mean-state static stability of a model’s troposphere. Factors like a greater rate of entrainment into convective updrafts, which reduces the buoyancy of convective plumes, is associated with a less stable troposphere. This decrease in stability occurs because the environment sits near thermodynamic thresholds determining convection onset. This work suggests that the response of the circulation – and the strength of the low cloud feedback – depends critically on these thresholds.

The Radiation-Subsidence and Stability-Subsidence mechanisms are likely to be strongest in regions along convective margins. Additionally, while we focus on relationships between the changes to low cloudiness and subsidence rate throughout, the dω_500d_/dT_s_ and dLCF_d_/dT_s_ relationship is likely strongly influenced by the effect of d*ω*_500d_/dT_s_ on dRH700_d_/dT_s_, whereby enhanced subsidence leads to a drier lower free troposphere.

**Fig. 9:**
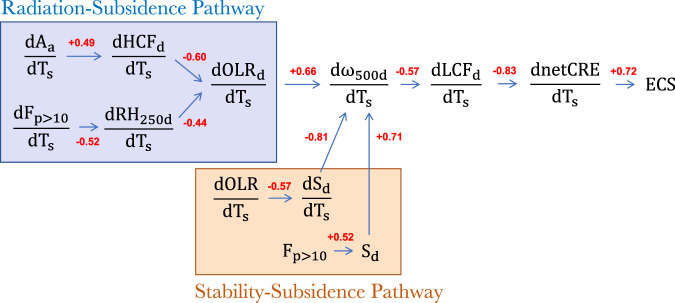
A summary of statistically significant correlations between various quantities comprising the Radiation-Subsidence and Stability-Subsidence Pathways. The correlations summarize the results shown in Figs. 2, 4, 6, and 7 among the CMIP6 multi-model ensemble. The Radiation-Subsidence Pathway is shown in blue, whereas the Stability-Subsidence Pathway is shown in orange. The direction of the arrows signifies suggested pathways of causality. Red values are Pearson correlation coefficients. All correlations are statistically significant at the 95% confidence level.

A summary of the correlations among variables connecting deep convection to the low cloud feedback and the intermodel spread in ECS through the Radiation-Subsidence and Stability-Subsidence Pathways can be seen in Fig. 9. Aside from the high correlations among dnetCRE/dT_s_, dLCF_d_/dT_s_, and ECS, which motivated this study, correlations are generally highest among terms explaining changes to d*ω*_500d_/dT_s_ and among the link between d*ω*_500d_/dT_s_ and dLCF_d_/dT_s_. Correlations among terms within the umbrella of a given pathway are lower, suggesting that there are multiple factors contributing to upper tropospheric changes that would modify the subsidence rate and subsequently the low cloud feedback.

**Fig. 10:**
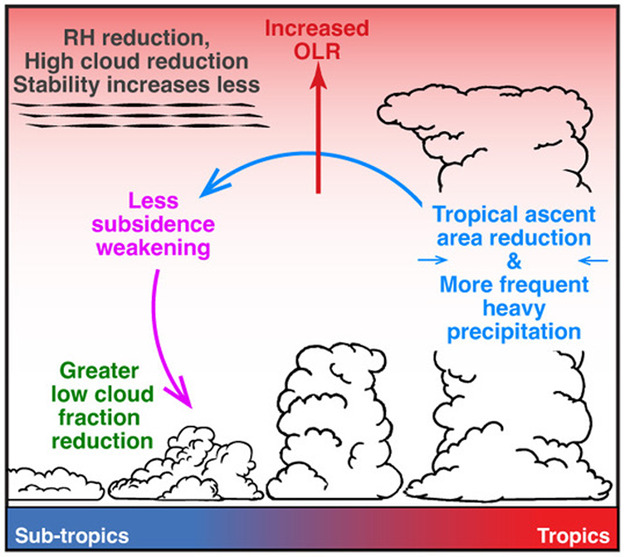
A summary schematic of the Radiation-Subsidence and Stability-Subsidence Pathways. A summary schematic illustrating how changes to deep convection link to the low cloud feedback by modifying upper tropospheric properties that affect the subsidence rate.

Overall, robust evidence among the multiple model ensembles examined in this study suggests that the strength of the low cloud feedback is intimately related to changes in deep convection through their effects on the overturning circulation. These relationships are depicted schematically in Fig. 10. Most notably, evidence suggests that a reduction of high cloudiness and enhanced UT drying (negative longwave feedback) leads to a net positive cloud feedback in high ECS models by contributing to a reduction in low cloudiness (positive shortwave feedback).

Many opportunities exist for future work exploring cloud-circulation interactions and the proposed mechanisms. First, the extent to which these mechanisms are acting in the real world is unknown, and thus trying to examine causal relationships between circulation, high clouds, and low clouds in observations will be a promising subject of future work. Linking the proposed pathways to changes in SST patterns and the “pattern effect” – the dependence of outgoing radiation to space on the spatial pattern of surface warming^63–70^ – would also be illuminating. Finally, improving our understanding of what physically drives differences in tropical ascent area, as well as frequency and intensity changes to heavy precipitation, is critical to our improved understanding of cloud-circulation coupling. New targeted model intercomparison studies, theoretical explorations of the physics controlling tropical ascent area, systematic examination of precipitation efficiency changes, examining the response of convective organization to warming, and idealized simulations of the tropical atmosphere would all drive significant progress towards this goal. Additionally, the extent to which identification of the Radiation-Subsidence and Stability-Subsidence pathways may permit constraints on ECS remains unanswered. Constraining the response of circulation to warming using observational estimates of static stability in the present climate, however, may be one potentially promising avenue.

## Methods

### Models

For our CMIP6 model analysis, we use the historical and Shared Socio-Economic Pathway 5 (SSP5-8.5) runs. SSP5-8.5 has a radiative forcing of 8.5 Wm^−2^ by the end of the 21^st^ century. Our analysis compares end-of-century mean quantities taken from 2086–2100 to historical mean quantities averaged from 2000–2014. ECS values are taken from the supplementary material of Zelinka et al.^2^ and from supplemental information from Hausfather et al.^71^ (10.1038/d41586-022-01192-2*)*. All data for composite mapping has been regridded to 2 × 2.5 degrees for our analysis.

The CMIP6 models used in our study are as follows: CAMS-CSM1-0, NorESM2-MM, NorESM2-LM, MIROC6, GFDL-ESM4, GISS-E2-1-G, FGOALS-g3, MPI-ESM1-2-HR, FGOALS-f3-L, BCC-CSM2-MR, MPI-ESM1-2-LR, GISS-E2-1-H, MRI-ESM2-0, CMCC-CM2-SR5, CMCC-ESM2, ACCESS-ESM1-5, GFDL-CM4, TaiESM1, ACCESS-CM2, CESM2-WACCM, NESM3, CNRM-ESM2-1, CNRM-CM6-1, CESM2, UKESM1-0-LL, and CanESM5. All models with output available for the analyzed variables were used in this analysis with a few exceptions. For example, published ECS values were not available at the time of analysis for CAS-ESM2-0 and E3SM-1-1-ECA. KACE-1-0-G and E3SM-1-0 were also excluded from the analysis because they proved to be significant outliers in static stability quantities for reasons that could not be explained (dS_d_/dT_s_ values 2-3 standard deviations away from the next nearest values in the ensemble).

Perturbed physics ensemble simulations are performed with the Community Atmosphere Model version 5.3 in the modified Zhang-McFarlane convection scheme^72,73^. The stratiform fall speed of ice is perturbed to be 350 s^−1^ and 1400 s^−1^ ^49^. Entrainment is perturbed from 0.08 to 1.5 km^−1^ (default is 1 km^−1^).

### Definitions

Low cloud fraction is the maximum at any given level between 600–1000 hPa (assuming maximum overlap), and high cloud fraction is taken as the maximum from 100–250 hPa. The pressure velocity at 500 hPa (*ω*_500_) is used to calculate tropical mean circulation and associated changes. Tropical ascent area (A_a_) is calculated by taking the mean of the fraction of grid boxes with *ω*_500_ < 0 hPa day^−1^ in a given month over the specified time periods noted above. Tropical descent area, which is used to determine the subsidence strength in descent regimes (ω_500__d_), is computed by taking the mean of the fraction of grid boxes with *ω*_500_ > 0 hPa day^−1^ in a given month.

The dominant energy balance of the subtropical atmosphere can be derived from the thermodynamic energy equation

3 $$\frac{\partial T}{\partial t}+u\frac{\partial T}{\partial x}+v\frac{\partial T}{\partial y}+\omega \left(\frac{\partial T}{\partial P}-\frac{{RT}}{P{c}_{p}}\right)={F}_{{net}}$$

in the absence of strong diabatic heating where

4 $$S=-\frac{T}{\theta }\frac{\partial \theta }{\partial P}=-\frac{\partial T}{\partial P}+\left(\frac{R}{{C}_{p}}\right)\frac{T}{P}$$

In the subtropics in the annual mean, $\frac{\partial T}{\partial t}\,\approx\, 0$, and $\left(u\frac{\partial T}{\partial x}+v\frac{\partial T}{\partial y}\right)\,\approx\, 0$, so that

5 $$-\omega S={F}_{{net}}$$

When ${F}_{{net}}$ is signed positive for column-integrated heating, we get Eq. 1 in the main text, where F_atm,d_ is signed positive for atmospheric cooling.

For the quantities used in Fig. 8c-d, the boundary layer is defined as the layer between 1000–900 hPa, and the lower free troposphere is defined as the layer between 900–500 hPa. Lower tropospheric instability is first computed as the difference between boundary layer averaged equivalent potential temperature (${\theta }_{{ebl}}$) and the lower free tropospheric saturation equivalent potential temperature (${\theta }_{{eL}}^{*}$), and then normalized by ${\theta }_{{eL}}^{*}$. This quantity is then multiplied by 340 K to be expressed in units of K. Similarly, subsaturation is computed as the difference between ${\theta }_{{eL}}^{*}$ and the lower free tropospheric equivalent temperature (${\theta }_{{eL}}$), and then normalized by ${\theta }_{{eL}}^{*}$. This quantity is then also multiplied by 340 K to be expressed in units of K. Detailed derivations of these quantities are available in^55,60^.

### Reporting summary

Further information on research design is available in the Nature Portfolio Reporting Summary linked to this article.

## Supplementary information

Supplementary Information

Reporting Summary

## Data Availability

All of the CMIP6 historical, SSP5-8.5, and CFMIP model output used in this analysis can be freely accessed through the Earth System Grid Federation (https://esgf-node.llnl.gov/projects/cmip6/). Data from the CAM5.3 PPE experiments can be accessed through http://schiro.evsc.virginia.edu/index.php/data/.

## Code availability

Analysis scripts are available through http://schiro.evsc.virginia.edu/index.php/data/.
