# Supplementary material for: Model spread in tropical low cloud feedback tied to overturning circulation response to warming
Source: Nat Commun. 2022 Nov 19;13:7119. doi: 10.1038/s41467-022-34787-4 (PMC9675744; doi:10.1038/s41467-022-34787-4)
Supplement: Supplementary file 1 — Supplementary Information [file 41467_2022_34787_MOESM1_ESM.docx]

Supplementary Information

**Model spread in tropical low cloud feedback tied to overturning circulation response to warming**

Kathleen A. Schiro^1*^, Hui Su^2,3,4^, Fiaz Ahmed^2,3^, Ni Dai^2^, Clare E. Singer^5^, Pierre Gentine^6^, Gregory S. Elsaesser^7,8^, Jonathan H. Jiang^9^, Yong-Sang Choi^10^, and J. David Neelin^2,3^

^1^Dept. of Environmental Science, University of Virginia, Charlottesville, VA, USA

^2^Joint Institute for Research in Earth Systems Science and Engineering, University of California, Los Angeles, Los Angeles, CA, USA

^3^Department of Atmospheric and Oceanic Sciences, University of California, Los Angeles, Los Angeles, CA, USA

^4^Department of Civil and Environmental Engineering, The Hong Kong University of Science and Technology, Hong Kong SAR, China

^5^Department of Environmental Science and Engineering, California Institute of Technology, Pasadena, CA, USA

^6^Earth and Environmental Engineering, Columbia University, New York, NY, USA

^7^Department of Applied Physics and Applied Mathematics, Columbia University, New York, NY, USA

^8^NASA Goddard Institute for Space Studies, New York, NY, USA

^9^Jet Propulsion Laboratory, California Institute of Technology, USA

^10^Department of Climate and Energy Systems Engineering, Ewha Womans University, Seoul, South Korea

*Corresponding author: Kathleen A. Schiro, Dept. of Environmental Sciences, 291 McCormick Road, Charlottesville, VA 22904 | Email: [kschiro@virginia.edu](mailto:kschiro@virginia.edu)


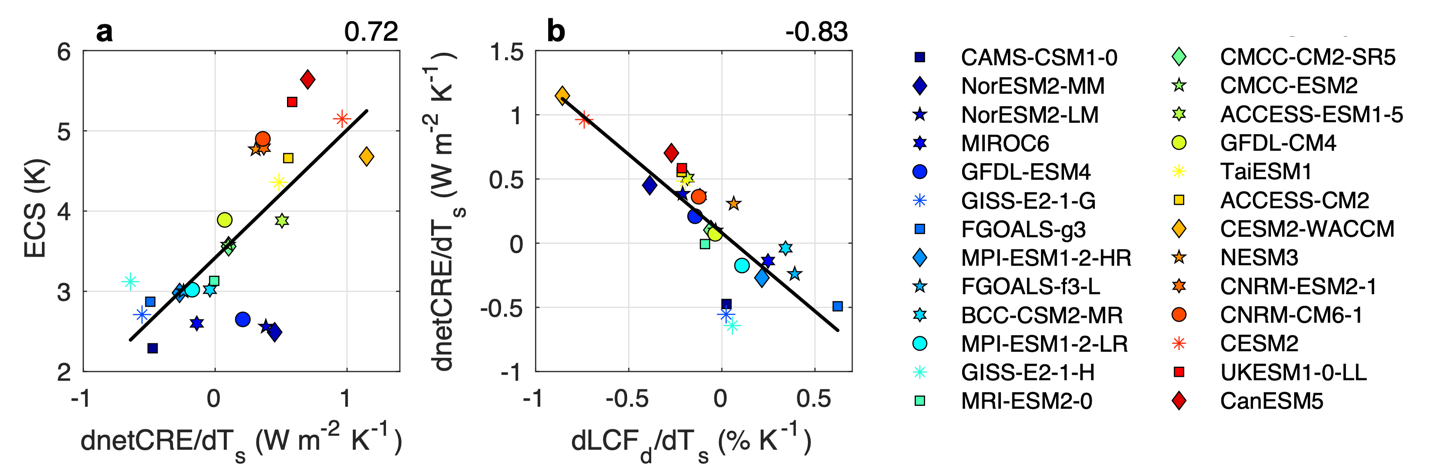


**Supplementary Figure 1: The change in low cloud fraction in tropical descent regions explains 69% of the variance in the change in tropical net cloud radiative effect, which explains 51% of the variance in climate sensitivity.** The relationships between (a) the change in net cloud radiative effect (dnetCRE/dT_s_; W m^-2^ K^-1^) and equilibrium climate sensitivity (ECS; K^-1^) and (b) the change in descent region low cloud fraction (dLCF_d_/dT_s_; % K^-1^) and dnetCREs/dT_s_. Changes are calculated as the difference between 2086-2100 (SSP5-8.5) and 2000-2014 (historical). Values in the upper right are the Pearson correlation coefficients. 26 CMIP6 models are included in the analysis. The color scale from blue to red reflects increasing ECS values. All relationships shown are tropical averages (30^o^S-30^o^N) including both land and ocean points.


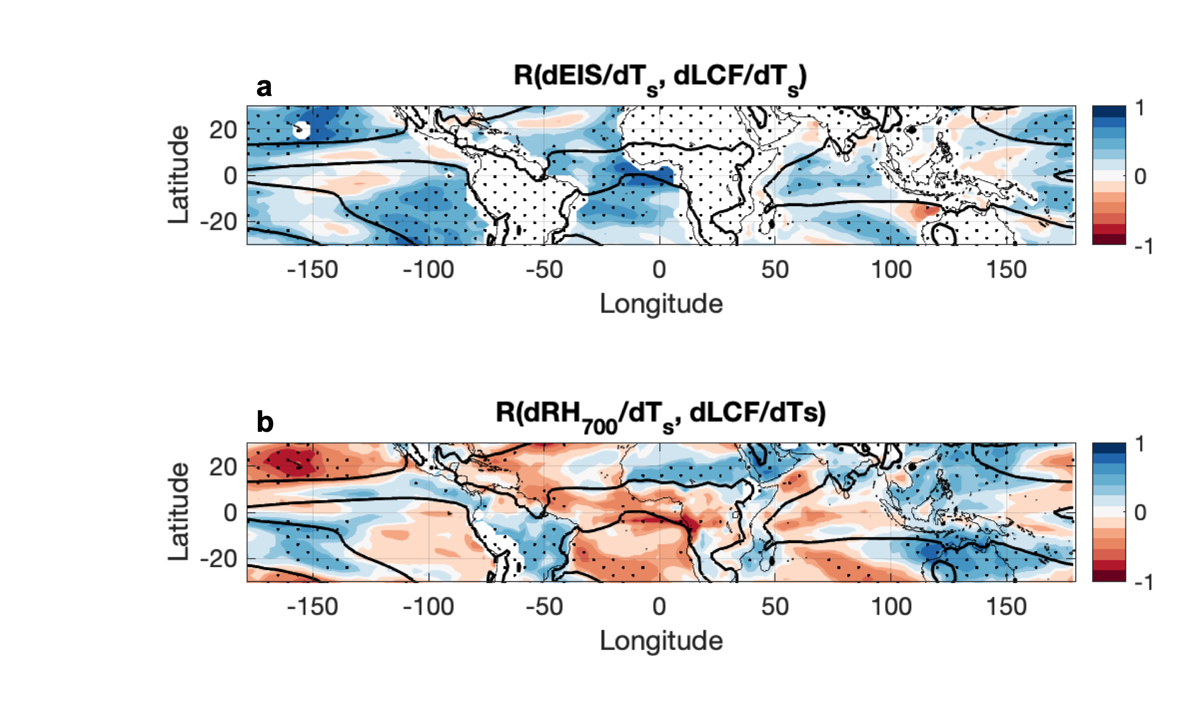


**Supplementary Figure 2: Correlation maps illustrating where intermodel differences in changes in estimated inversion strength and free tropospheric relative humidity relate most closely to intermodel differences in changes in low cloud fraction.** The Pearson correlation coefficients between (a) the change in estimated inversion strength (dEIS/dT_s_; K K^-1^) and the change in low cloud fraction (dLCF/dT_s_; % K^-1^) and (b) the change in 700 hPa relative humidity (dRH_700_/dT_s_; % K^-1^) and dLCF/dT_s_ (% K^-1^). Stippling indicates that the relationships are statistically significant at 95% (p < 0.05) among the 26 models used in the study.

**Supplementary Figure 3: Intermodel differences in circulation strength changes are significantly correlated with intermodel differences in outgoing longwave radiation changes throughout most regions of the tropics.** Same as Figure S3 except for the Pearson correlation coefficients between (a) the change in pressure velocity at 500 hPa (d$\omega$_500_/dT_s_; hPa day^-1^ K^-1^) and the change in outgoing longwave radiation (dOLR/dT_s_; W m^-2^ K^-1^).


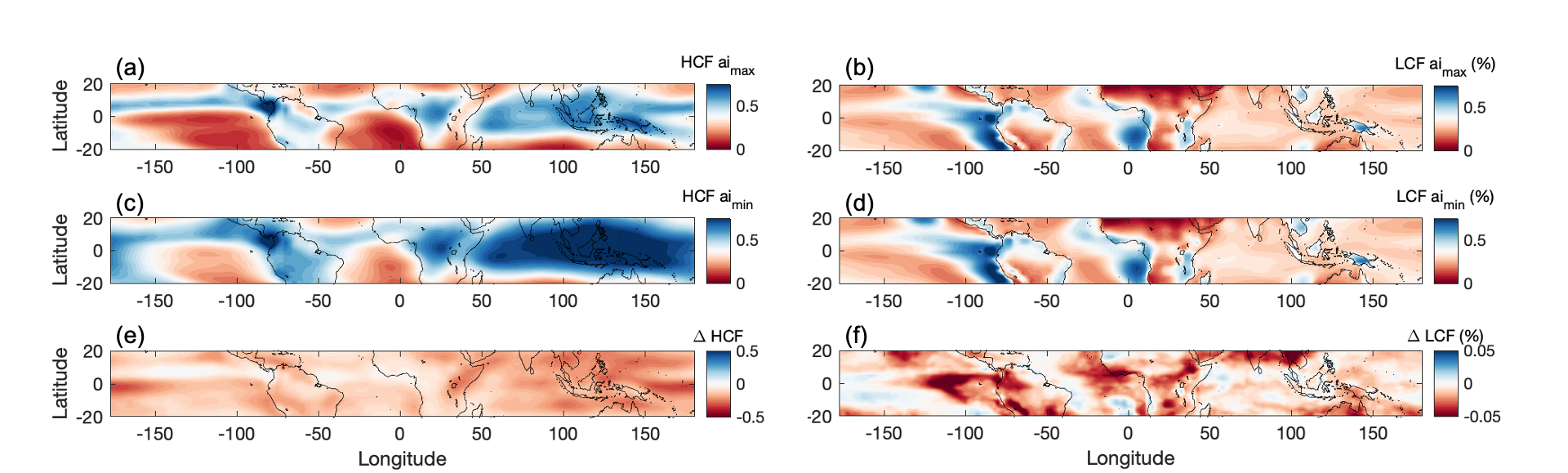


**Supplementary Figure 4: An increase in stratiform ice fall speed leads to a decrease in high cloud fraction across the tropics, which corresponds to a decrease in low cloud fraction across the tropics.** (left column) The mean high cloud fraction (HCF) in the Community Atmosphere Model version 5.3 (CAM5.3) with the fall speed of stratiform ice modified to be (a) 1400 s^-1^ (double the default value of 700 s^-1^) and (c) 350 s^-1^ (half of the default value). The difference between panels (a) and (c) is shown in (e). (right column) The mean low cloud fraction (LCF) in CAM5.3 with the fall speed of stratiform ice modified to be (b) 1400 s^-1^ (double the default value of 700 s^-1^) and (d) 350 s^-1^ (half of the default value). The difference between panels (b) and (d) is shown in (f).

**Supplementary Figure 5:** **Disabling the interaction between outgoing longwave radiation and clouds in CNRM-CM6-1 results in a decrease in lower tropospheric stability and corresponding decrease in low cloud fraction throughout the tropics, particularly in stratocumulus regions**. Differences in (a) outgoing longwave radiation (OLR; W m^-2^), (b) lower tropospheric stability (LTS; K), and (c) low cloud fraction (LCF; %) are shown. The control climate (lwon) is subtracted from an atmosphere-only experiment in which longwave cloud radiative effects were disabled (lwoff).
